# Property Variation in Wavelength-thick Epsilon-Near-Zero ITO Metafilm for Near IR Photonic Devices

**DOI:** 10.1038/s41598-020-57556-z

**Published:** 2020-01-20

**Authors:** Jimmy H. Ni, Wendy L. Sarney, Asher C. Leff, James P. Cahill, Weimin Zhou

**Affiliations:** CCDC Army Research Laboratory, Sensors and Electron Devices Directorate, 2800 Powder Mill Road, Adelphi, MD 20783 USA

**Keywords:** Metamaterials, Metamaterials

## Abstract

Thin indium tin oxide (ITO) films have been used as a medium to investigate epsilon-near-zero (ENZ) behavior for unconventional tailoring and manipulation of the light-matter interaction. However, the ENZ wavelength regime has not been studied carefully for ITO films with thicknesses larger than the wavelength. Thick ENZ ITO film would enable the development of a new family of ENZ-based opto-electronic devices that take full advantage of the ENZ behavior. Here, we demonstrated wavelength-thick ITO films reaching the ENZ regime around a wavelength of 1550 nm, which permit the design of such devices operating in the common optical telecommunications wavelength band. We discovered that the permittivity of the film was non-uniform with respect to the growth direction. In particular, after annealing at a sufficiently high temperature, the real part of the permittivity showed a step change from negative to positive value, crossing zero permittivity near the middle of the film. Subsequently, we conducted comprehensive microanalysis with X-ray diffraction, transmission electron microscopy (TEM) and energy dispersive X-ray spectroscopy (EDS) to investigate the correlation of the permittivity variation with variations in the ITO crystallite morphology and relative concentrations of different atom species. The result of this study will allow us to design a new family of opto-electronic devices where ITO can be used as the cladding that guides light within an air-core waveguide to provide a new platform to explore ENZ properties such as environment insensitivity, super-coupling, and surface avoidance. We have also provided a comprehensive method to determine the permittivity in a non-uniform ENZ material by using an advanced physical model to the fit experimental data.

## Introduction

Materials where the permittivity, ε, approaches zero (ENZ) have attracted substantial interest in the academic community due to numerous interesting phenomena predicted to occur within this regime. Namely, as the permittivity approaches zero, the material is predicted to exhibit enhanced optical nonlinearity^1,2^ and EM waves propagating within will have increased wavelength and phase velocity^3^. These properties have inspired research into a variety of novel devices including sub-wavelength and high light-matter-interaction electro-optic modulators^4^, deeply sub-wavelength ENZ-filled channels for unconventional routing of electromagnetic (EM) waves^3,5–7^, and perfect absorbing layers^8^.

ENZ conditions have been demonstrated at wavelengths across the electromagnetic spectrum^7,9–13^, occurring, for example, near the plasma frequency of a material’s free carriers, at the band edge of a waveguide, or as an average effect of metal-dielectric layer stacks^3^. In general, the ENZ regime occurs in a wavelength range where both the real part and the imaginary part of the permittivity are close to zero. Materials with ENZ in the wavelength range of 1525 nm – 1565 nm are of particular interest for applications in optical telecommunications, RF-photonics, and integrated silicon photonics. Thin films with ENZ wavelengths in this spectral region have been studied in transparent conducting oxides, such as indium tin oxide (ITO) and aluminum-doped zinc oxide (AZO)^12,13^.

Among these materials, ITO has shown particular promise, because of its potential for low-cost fabrication and compatibility with silicon-based integrated photonics. ITO is widely used in commercial semiconductor integrated circuits. Therefore, it is an attractive material to use for developing ENZ based optoelectronic devices due to its compatibility with well-established integrated photonics fabrication processes. In addition, annealed ITO has been found to have a faster recombination time than many semiconductors, since low-temperature (below 400 C) annealing can enable ultrafast recombination. Operated in the ENZ regime, larger changes in the reflection for a fixed change in the refractive index can be produced at technologically important telecom wavelengths^14^.

A thermal-annealing process allows the formation of a self-assembled ITO metamaterial to achieve the ENZ condition at the desired 1550 nm optical telecommunications band. In that frequency band, the permittivity of the ITO can be described by the Drude model, where incident light interacts with the free carriers in the material, and their response becomes resonant at the plasma frequency^15,16^. Based on this model, the real part of the permittivity crosses zero at the frequency ***ω***_0_, which is given by

$${{{\boldsymbol{\omega }}}_{0}}^{2}=[{q}^{2}/({{\boldsymbol{\varepsilon }}}_{0}{{\boldsymbol{\varepsilon }}}_{\infty }{m}^{\ast })]N\mbox{--}1/{\tau }^{2},$$

where *q* is the elementary charge, ***ε***_0_ is the vacuum permittivity, ***ε***_∞_ is the permittivity at high frequency, m^*^ is the effective electron mass, *N* is the electron density, and *τ* is the mean scattering time of the electrons. Since the elementary charge, effective mass, and vacuum and high-frequency permittivities are approximately constant, ***ω***_0_ is predominantly determined by the electron density and scattering time.

The annealing process for ITO films alters ***ω***_0_ in a complicated fashion. Various material parameters contribute to the carrier density and scattering time, including the oxygen vacancy density, which affects both the carrier density and the scattering time^4,17–22^, and the crystal grain size, which affects the scattering time^21^. Low-oxygen deposition and post-deposition annealing of the ITO films have been demonstrated to alter the plasma frequency by altering material parameters such as these^17,20,21,23,24^. However, it remains an open question how to best process an ITO film in order to control each parameter such that the plasma frequency is precisely controlled and uniform. One limiting factor in achieving this level of control has been the relative lack of microanalysis of ENZ ITO self-assembled metamaterial-films.

Moreover, to the authors’ knowledge, in all the previously reported ENZ ITO film studies, the thickness of these films were significantly less than the wavelength, which limits the design and realization of photonic devices that can fully benefit from the ENZ property. Thick films of ENZ material, where the thickness is on the order of the wavelength would enable devices where the ENZ material acts as the cladding that guides light within an air-core waveguide. This architecture would enable a new family of devices that take advantage of the ENZ material’s novel properties in new ways. For example, an air-core resonator embedded in ENZ ITO cladding material may reduce the environmental insensitivity. Notably, in this architecture, if the imaginary part of the permittivity is sufficiently high, the incident light will suffer intolerably high propagation loss. However, as we show in the Supplementary Material, if the imaginary part of permittivity is less than 0.6, the waveguide simulation indicated that the light will be index guided in the air-core and have relatively low propagation loss. This loss could be reduced in a disk resonator design since it has one less ITO sidewall. Hence, it is crucial to develop ENZ material that has permittivity that meets those criteria at the wavelength of interest.

In this article, we studied the permittivity and microstructure of several wavelength-thick ITO films annealed at temperatures ranging from 250 C to 400 C. The permittivity varied as a function of the depth in each sample. Those annealed at 250 C and 300 C had slowly varying permittivity depth profiles with ENZ wavelengths of 1530 and 1450 nm, respectively. These samples may be suitable for standard waveguides and device designs. In the samples annealed at 350 C or greater, the permittivity depth profile showed a sharp transition mid-way through the film; at a wavelength of 1550 nm, the bottom portion of the film had negative permittivity, whereas the top portion had positive permittivity, and a thin layer in between had permittivity near zero. These samples may be suitable for specialized devices that take advantage of this steep transition.

Furthermore, to investigate the possible physical mechanisms that contributed to the permittivity depth profiles, we performed microanalysis on one high-temperature-annealed and one low-temperature-annealed sample and compared them with an as-grown ITO sample. In the high-temperature-annealed sample, there was a distinct change in crystal grain size and morphology that occurred approximately mid-way through the ITO film. This feature is not present in the low-temperature-annealed sample. This result may indicate that a reduced scattering time in the top of the high-temperature-annealed film contributed to the observed permittivity profile. Additionally, in the high-temperature-annealed sample, the oxygen concentration experienced an abrupt increase mid-way through the film depth that was not observed in the low-temperature-annealed sample. This result may indicate that a reduction of oxygen vacancies in the top of the high-temperature-annealed film contributed to the observed permittivity profile. Although our analysis was unable to determine quantitatively how much each physical mechanisms contributed in the permittivity profile, the results will guide future research to control the permittivity of the ITO film throughout its depth profile. The result also provided a method to determine the epsilon profile in an anisotropic ITO film.

## Results

### Permittivity of thick ITO films

**Figure 1:**
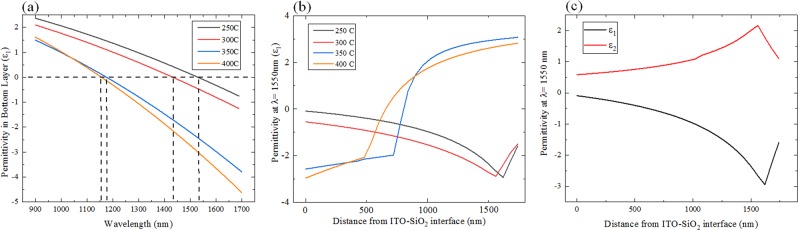
(**a**) Permittivity as function of wavelength for 4 different annealing temperatures; (**b**) Real parts of the permittivity depth profile of 2 µm-thick ITO film for different annealing conditions of ITO metafilms; (**c**) Real and imaginary parts of the permittivity depth profile of 250 °C annealed ITO film.

We deposited the ~2 µm thick ITO films via pulsed DC sputtering onto a SiO_2_ layer which sat on top of a Si substrate. This layer stack, shown in Fig. 1, emulates a typical layer stack used for integrated silicon photonic devices (SOI). In order to create samples with different plasma frequencies, we annealed samples diced from this film stack for 10 minutes at temperatures ranging from 250 C to 400 C in N_2_ atmosphere. Then, we performed variable angle spectroscopic ellipsometry (VASE) to measure the permittivity of the ITO layer in each sample (see supplementary information for more details). We found that for all annealing temperatures, the permittivity in the ITO layer was not constant with respect to the depth. At lower annealing temperatures, the samples’ permittivity depth profile showed a relatively small amount of non-uniformity, which is qualitatively consistent with the behavior reported for thin ITO films. However, at higher annealing temperatures, a sharp transition region appeared midway through the depth of the ITO film. Above this transition region—i.e., near the top of the ITO film—the real part of the permittivity was positive, whereas below the transition region—i.e., near the bottom of the ITO film—the real part of the permittivity was negative.

Figure 1(a) shows the wavelength dependence of the real part of the permittivity measured in the ITO films near the substrate interface. The samples annealed at 250 C and 300 C had ENZ wavelengths near the telecommunications band, at 1530 nm and 1450 nm, respectively. The samples annealed at 350 C and 400 C had significantly shorter plasma wavelengths, at 1360 nm and 1340 nm, respectively. These results are in agreement with previous observations of thin ITO films, which show that as the annealing temperature increased, the plasma wavelength became shorter. We note that the variable angle spectroscopic ellipsometry technique used to measure the permittivity results in a significant uncertainty in determining the ENZ wavelength. This limitation arose from three major factors: (1) the lack of a well-defined permittivity model for ITO, (2) large film-thickness and non-uniformity that led to a complicated reflectance profile, and (3) the difficulty in directly measuring depth-dependent material parameters, such as the carrier scattering time and resistivity of the ITO. Nevertheless, the measured permittivity indicates that in the 250 C- and 300 C-annealed samples, the ENZ regime was reached at optical telecommunications wavelengths.

To illustrate clearly the non-uniformity of the permittivity in the ITO layer, we chose a wavelength of 1550 nm at which to examine the measured data. Figure 1(b) shows the real part of the measured permittivity at this wavelength as a function of the distance from the SiO_2_-ITO interface. For the samples annealed at 250 °C and 300 °C, the real part of the permittivity varies slowly, ranging from −0.1 to −3.0 and from −0.5 to −2.9, respectively. By contrast, for the samples annealed at 350 °C and 400 °C, the real part of the permittivity is highly non-uniform. In both samples, the real part of the permittivity is negative at the interface with the substrate and increases slowly until it reaches a threshold distance. Beyond this depth, the permittivity increases sharply and becomes positive. Figure 1(c) shows both real and imaginary parts of the ITO film annealed at 250 °C. As we show in a later section, this behavior may be the result of depth-dependent variations in both or either the crystal grain size/morphology or oxygen vacancy density.

### Non-uniform permittivity of the ITO film

In the previous section, we showed that for the samples annealed above a threshold temperature, there was a sharp transition in the permittivity depth profile, whereas no such transition existed for samples annealed below a threshold temperature. To gain more insight into this behavior, we conducted several microanalysis experiments on three of our ITO samples: as-deposited, annealed at 250 °C, and annealed at 350 °C. Specifically, we examined the crystallinity and texture using x-ray diffraction (XRD), the crystallite morphology using cross-sectional transmission electron microscopy (TEM), and the chemical composition using energy dispersive x-ray spectroscopy (EDS). The XRD revealed that the as-deposited sample was predominately amorphous, while the annealed samples were polycrystalline with no dominant crystal orientation. The TEM revealed that the crystal grains in the high-temperature-annealed sample changed size and shape partway through the film’s thickness, whereas the crystal grains in the low-temperature-annealed samples showed no such change (see supplementary information for more details). The EDS further showed that in the high-temperature-annealed sample, the oxygen concentration increased sharply partway through the film, whereas the low-temperature-annealed sample showed no such change. These results indicate that the crystal grain morphology and oxygen vacancy density may contribute significantly to the permittivity of the ITO film.

**Figure 2:**
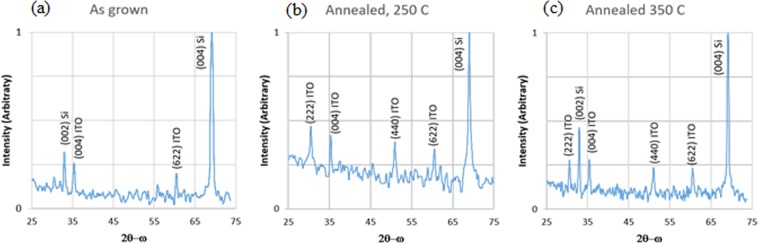
XRD spectra of: (**a**) as-deposited, (**b**) 250 °C -annealed, and (**c**) 350 °C -annealed samples.

We collected high-resolution 2θ-ω x-ray diffraction scans with a Panalytical X’Pert Pro system using a 1D PIXcel detector in scanning mode. The x-ray scan plots have with a logarithmic intensity scale with arbitrary units on the y-axis over a 50° range in 2θ on the x-axis. Reference powder diffraction data shows that the strongest x-ray peak for ITO is (222). The other three strong peaks are the (004), (440) and (622) peaks with 30.5, 36.8, and 26.9% of the intensity of the (222) peak. The XRD data in Fig. 2 reveal that the as-deposited sample was predominantly amorphous, while the annealed samples were polycrystalline, with no dominant orientation.

**Figure 3:**
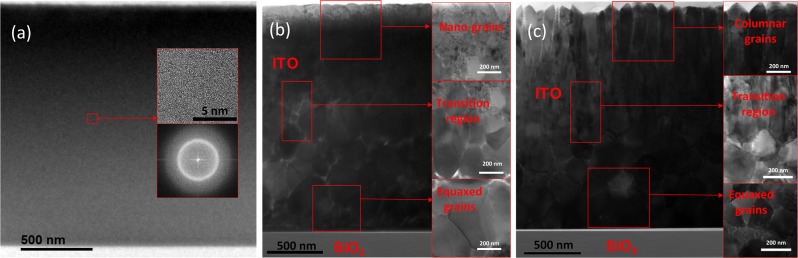
TEM images of (**a**) as-deposited, (**b**) 250 C-annealed, and (**c**) 350 C-annealed ITO films.

For the as-deposited sample, Fig. 2a shows the strongest peak corresponds to the (400) orientation of the crystalline Si substrate. There were two weak peaks corresponding to the r(004) and (622) orientations of the ITO film. As shown in the inset of Fig. 3a, the TEM diffraction pattern confirms that the as-deposited ITO film consists of a diffuse orbit, indicating that the two ITO-related peaks are due to small crystallites. Because the TEM sample crystallizes quickly due to localized heating from the electron beam, we could not extensively examine the orientation of the small polycrystallites without affecting the sample.. For the sample annealed at 250 C, (Fig. 2b) we find ITO peaks corresponding to the (222), (004), (440), and (662) orientations, respectively. As compared to the reference powder diffraction data, the (222) peak is not as strong relative to the other three reflections. This indicates that the grain orientations are not completely random, but all four primary reflections are present. Figure 2c is the XRD for the sample annealed at 350 C, which is similar to that seen in for the sample annealed at 250 C. although the (222) peak is slightly weaker.

**Figure 4:**
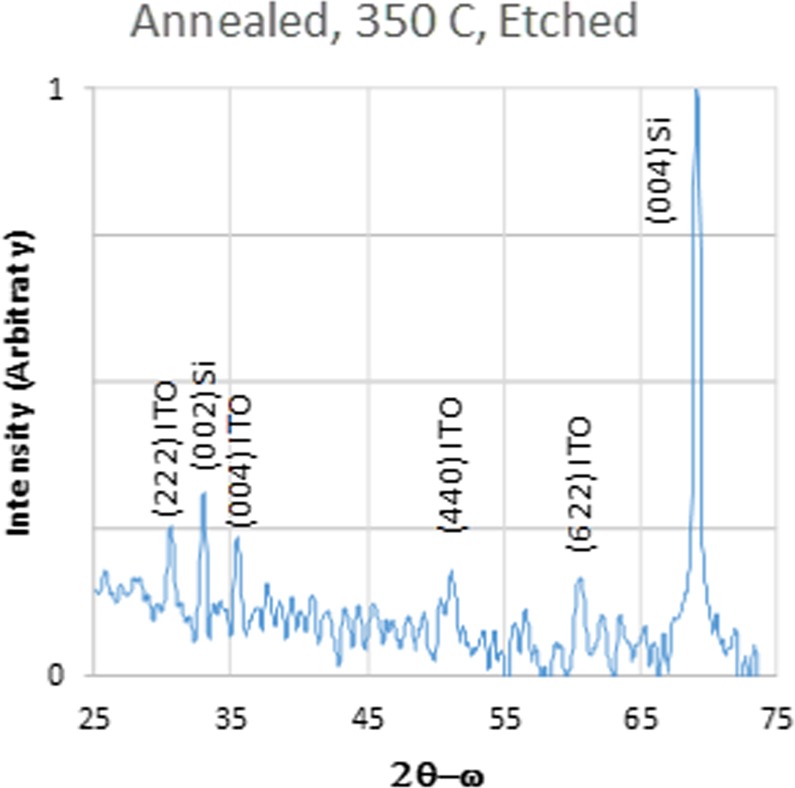
X-ray diffraction scan of the 350 C-annealed sample after the top 1 µm of the ITO layer was etched away. The similarity between this spectrum and the spectrum in Fig. 2c indicates that there is no dominant either texture in the top or bottom half of the film.

Further XRD analysis showed that the crystal structure and lack of a predominant orientation was consistent throughout the 350 C-annealed ITO film, despite our observations that the permittivity and, as described later, the morphology and atomic composition of the 350 C-annealed ITO film displayed depth-dependence. We etched off the top 1 µm of ITO from the 350C-annealed ITO sample and characterized the remaining ITO film by XRD. The results are shown in Fig. 4. The same peaks were observed although the (004) peak is slightly weaker, suggesting that the top portion of the film had a preference to align along that direction. Hence, although the crystal grain size and shape changed throughout the 350 C-annealed ITO film, the XRD data shows that the crystal structure remained the same throughout the film.

The TEM analysis showed that in the 350 C-annealed sample there was distinct change in the crystal grain morphology approximately halfway through the ITO layer, while in the 250 C-annealed film, the crystal grain morphology was consistent throughout the ITO layer. Figure 3(a–c) show cross-sectional TEM images of the as-deposited, 250 C-annealed, and 350 C-annealed ITO samples, respectively. The low magnification images display the entire profile of the ITO film, from the SiO_2_ interface to the film surface. The as-deposited sample appears uniform throughout its depth profile. A TEM diffraction pattern (inset of Fig. 3(a)) shows that the as-deposited sample was amorphous. The 250 C-annealed sample was polycrystalline, with equiaxed crystal grains forming throughout the majority of the film as shown in Fig. 3(b). Near the surface and SiO_2_ interface, there were boundary layers of varying thickness that were composed of nano-grains. The equiaxed grains in the middle of the film had diameters from approximately 120 nm to 300 nm, and pores were visible in between some of these grains. The 350 C-annealed film was also polycrystalline, and the grain morphology varied across the film depth profile, as shown in the inset images of Fig. 3(c). The bottom of the film exhibited equiaxed grains, whereas the top of the film exhibited columnar grains that extended to the film surface. In contrast to the as-deposited film, which had a smooth surface, the surface roughness of the 350 C-annealed film was 104 nm due to faceting of the columnar grains. In between these regions was a transition region, where the equiaxed crystal grains met the bottom of the columnar grains. The equiaxed grains in the bottom layer had diameters from approximately 160 nm to 350 nm.

**Figure 5:**
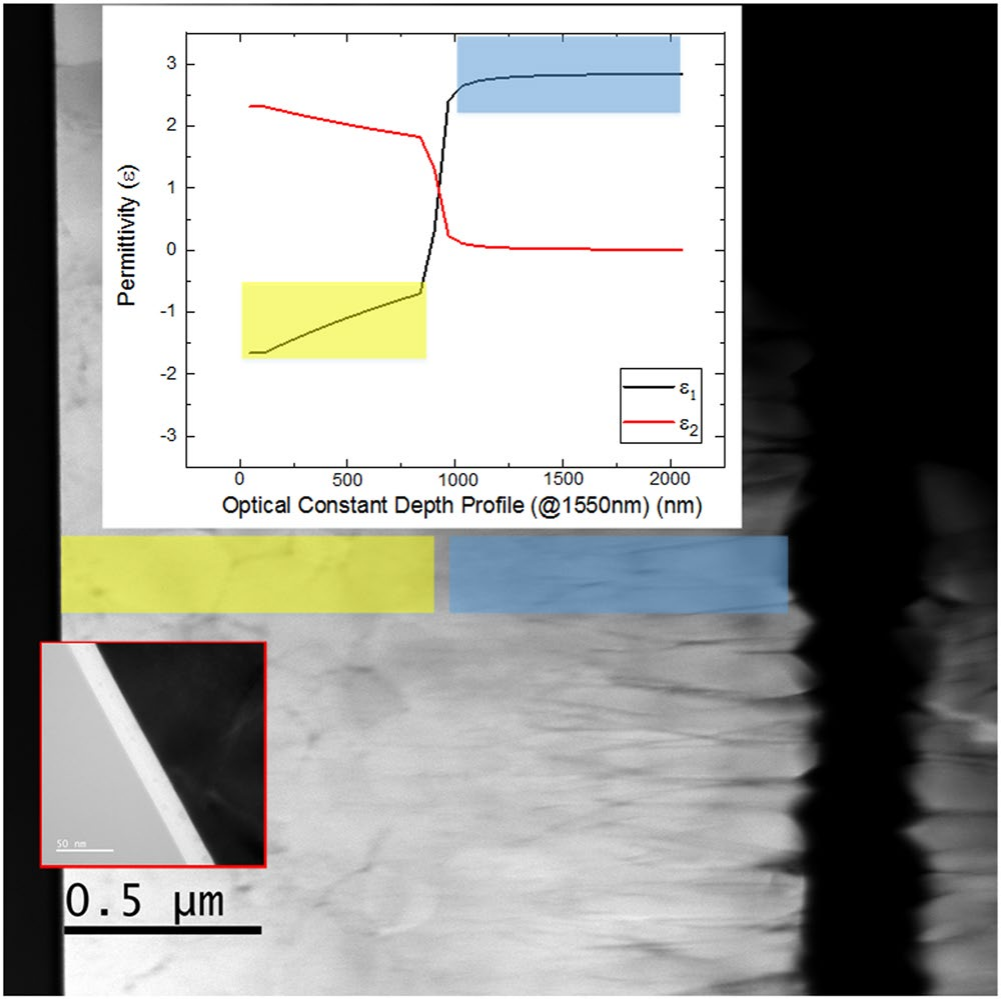
STEM-HAADF image of the annealed ITO film, in comparison with it optical property.

The depth at which we observed the permittivity change in the ellipsometry data corresponds to the depth at which we observed the crystal grain morphology change in the TEM image. To illustrate this point, Fig. 5 shows a scanning TEM high angle annular dark field (STEM-HAADF) image with the corresponding ellipsometry data overlaid. The yellow and blue overlays represent the correspondence of the local crystal grain with permittivity: the permittivity depth profile aligns well with the crystalline morphology change. STEM-HAADF imaging shows mass-thickness contrast, revealing that there is intergranular porosity throughout the film thickness, which is greatest toward the film surface, with a minimum in the middle of the film in the transition region.

**Figure 6:**
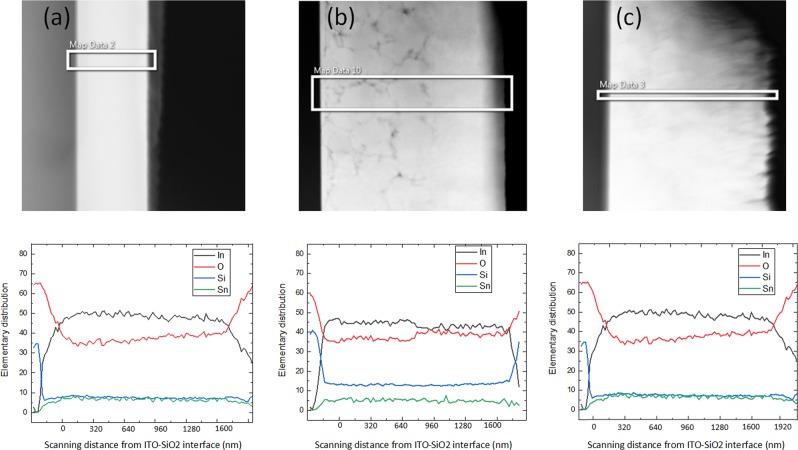
(Top): Cross-section image; (Bottom): EDS mapping of the ITO layer, atomic concentration (%) vs. ITO depth in (nm) from interface, in the **(a)** as-deposited, **(b)** 350 C-annealed, and **(c)** 250 C-annealed samples.

As shown in Fig. 6, the EDS analysis revealed several important details about the atomic composition of the ITO films, including that in the 350C-annealed sample, the oxygen concentration experienced a step increase near the middle of the ITO layer as shown in Fig. 6(b), while in the 250 C-annealed sample it varied slowly in Fig. 6(c). All of the ITO films analyzed contained a significant amount of Si in addition to the expected In, Sn, and O, which may have entered the film via diffusion from the silica layer. Moreover, each ITO film was oxygen-deficient, with the observed oxygen concentration significantly below the expected stoichiometric concentration of approximately 60%, and the ratio of O to In well below the expected value of approximately 1.8. Thin films of ITO are often reported in the literature to be oxygen-deficient in order to promote oxygen vacancies, which raise the carrier density. As shown in Fig. 6(b), in the 250C-annealed film, the concentrations of the In and O varied slowly throughout the layer, ranging from 50% to 43% and 33% to 40%, respectively. By contrast, in the 350C-annealed film, Fig. 6(c) shows the concentrations of In and O were approximately constant in the bottom half of the film at 45% and 35%, respectively. In the middle of the film, the In and O concentrations experienced a sharp transition, after which their concentrations converged to approximately 40%. Notably, the transition in concentration occurred at approximately the same depth as the transition in the permittivity of the same sample, which indicates that the two transitions may be related. While the oxygen vacancy concentration was not measured directly, the trend in oxygen concentration may indicate an analogous trend in the concentration of oxygen vacancies, which would influence the permittivity.

## Discussion

In this article, we fabricated and characterized wavelength-thick ITO films with ENZ wavelengths around 1500 nm, where we set the ENZ wavelength of each sample by changing the annealing temperature. The samples annealed at 250 C and 300 C displayed a permittivity that varied slowly with respect to film depth. Importantly, these samples could be used to design devices with ENZ cladding in the commonly used telecommunications wavelength range (see supplementary information for more details).

The samples annealed at 350 C and 400 C displayed a sharp transition in the permittivity approximately half-way through the depth of the ITO layer. This transition may be useful for specially designed devices that take advantage of such a sharp change in permittivity. Moreover, the permittivity change provided a convenient distinguishable feature that allowed us to identify clearly correlations between the microstructure of the film and its permittivity.

Prior studies of thin ITO films have identified several different mechanisms by which the material properties can affect the plasma frequency and, hence, the permittivity of the film. However, establishing which mechanism is dominant in a given ITO film has remained challenging and generally must be done on a case-by-case basis. The challenge is in some ways even greater in the case of thick films, because while the film varies significantly throughout its depth, it is difficult to measure the relevant parameters in a depth-resolved fashion. Consequently, although the microanalysis in this article revealed several trends that correspond to established mechanisms that affect the permittivity, more work is required to quantitatively conclude which mechanisms are dominant. Nevertheless, our microanalysis may point towards methods to control the sharp permittivity rise found in the high-temperature-annealed samples and the residual permittivity gradient in the low-temperature-annealed films. For example, in thin ITO films, it has been shown that increasing crystal grain size reduced the characteristic time associated with carriers scattering at the crystal grain boundaries, thereby shortening the plasma wavelength^21^. By comparison, we found that the top half of the 350 C-annealed ITO film was composed of columnar-shaped grains with widths ranging from ~80 nm to 200 nm and significant porosity between grains, while the bottom half of the ITO film was composed of larger, equiaxed grains with average diameters ranging from approximately 160 nm to 350 nm and lower porosity between grains. One possible explanation for the formation of the columnar grains is that the strain build-up during the crystallization may cause partial relaxation which results in preferential crystal growth in the vertical direction, therefore changing the shape of the crystal grains. These observations may indicate that the scattering time is shorter in the top half of the film than the bottom, so that the top half would have a longer ENZ wavelength, in agreement with the measured permittivity data. Hence, one possible strategy to mitigate the sharp permittivity change in the high-temperature-annealed ITO films is to prevent the formation of the pillar-shaped grains, so that the grain size is consistent through the high-temperature-annealed films.

Additionally, in thin ITO films, it has been shown that a decrease in the oxygen vacancies in the ITO crystal lattice can be associated with either a longer or shorter ENZ wavelength^4,17–21^. When the concentration of oxygen vacancies is above a critical value, the carriers have a high probability to scatter from the vacancy sites. In this regime, decreasing the vacancy concentration increases the mean scattering time and thereby shortens the ENZ wavelength. In addition to creating a scattering site, each oxygen vacancy contributes two free carriers; as a result, when the vacancy concentration is below the critical value, changes to the free carrier contribution may become the dominant mechanism to affect the plasma frequency and, therefore, affect the ENZ wavelength. In this regime, decreasing the concentration of oxygen vacancies leads to a lower concentration of free carriers, which lengthens the ENZ wavelength. In this work, we found that all the samples we analyzed were heavily oxygen-deficient. Although atomic concentration does not directly relate to the vacancy concentration, this result may indicate that these samples had a high density of oxygen vacancies. Nevertheless, in the top half of the 350 C-annealed film, where we measured a discrete step to longer ENZ wavelength, we also found that there was a discrete rise in the oxygen concentration in the top half of the film, which may correspond to a decrease in the vacancy concentration. This result may indicate that although the sample was oxygen depleted, there were sufficiently few oxygen vacancies that the carrier concentration effect was dominant. The mechanism causing the change in the oxygen content remains unclear. Future work should address these questions.

Our work demonstrates the fabrication of thick ITO films reaching the ENZ region at telecommunications wavelengths. Moreover, we show that with certain fabrication parameters, the permittivity of the ITO films have a strong depth dependence, which appears to be related to the microstructure of the ITO. We have demonstrated a method to accurately determine the optical property of an anisotropic thick ENZ ITOfilm by using a physical model to fit the experimental ellipsometery data. We have carried out extensive crystallinity and chemical composition study (TEM and EDS) to verify our model and confirm our result. This will further impact the metamaterial and nanophotonics community by providing a more accurate optical properties of ENZ ITO films, especially for films thicker than few 100’s of nm. These results open the door for the design of optical devices where the cladding is in the ENZ region, including devices that may take advantage of depth-dependent permittivity.

## Supplementary information

Supplementary information.
