# Supplementary material for: Property Variation in Wavelength-thick Epsilon-Near-Zero ITO Metafilm for Near IR Photonic Devices
Source: Sci Rep. 2020 Jan 20;10:713. doi: 10.1038/s41598-020-57556-z (PMC6971020; doi:10.1038/s41598-020-57556-z)
Supplement: Supplementary file 1 — Supplementary information. [file 41598_2020_57556_MOESM1_ESM.pdf]

Supplementary Materials

Jimmy H. Ni*, Wendy L. Sarney, Asher C. Leff, James P. Cahill, and Weimin Zhou*

CCDC Army Research Laboratory

Sensors and Electron Devices Directorate

2800 Powder Mill Road

Adelphi, MD 20783

Corresponding authors: J. Ni, jimmy.h.ni.civ@mail.mil; W. Zhou, weimin.zhou.civ@mail.mil

S1. Computational Modeling of Air-Core/ITO waveguide

Previous analyses of ENZ-clad dielectric-core waveguides has suggested intolerably high propagation loss [S1]. However, the existing analysis is limited. In this section, we present preliminary computational modeling that indicates that the waveguide geometry can be tailored to reduce propagation loss as low as 25 dB/mm, which would allow fabrication of waveguide devices up to 120 µm long with less than 3 dB insertion loss. This result indicates that the ENZ-clad air-core waveguide is a promising avenue for device design.

Computational modeling suggests that the design of ITO ENZ based waveguide structure for waveguide structure involves geometry management for predictable and optimized performance. Finite element analyses provide context for general, rule-based procedures for obtaining an ENZ-based waveguide system with desired performance, as an essential requirement for practical applications. As shown in Figure S1, the structural design of an air-core ENZ-cladding waveguide includes the vertical thickness restriction and the horizontal layout for loss control. The hollow-core (air-core) structure used in this case is to focus on the impact brought by ENZ cladding. The air-core thickness is determined by the vertical restriction of ITO layer with its material property and thickness.

The waveguide propagation loss depends strongly on the waveguide geometry. The relatively large imaginary part of the ITO effective index results in considerable penetration depth compared to other waveguide cladding materials. The physical dimension of the hollow-core waveguide structure, like the waveguide thickness and width, and the ITO cladding thickness play crucial roles in determining the propagation loss. Representative examples presented in Figure S1 builds up the 2-D cross-sectional waveguide model of an ENZ-cladding hollow-core structure. The simulated air core thickness was set to be 4 µm, waveguide width is 10 µm. The ENZ cladding was 10 µm in thickness and 20 µm in width. A ridge (air) structure was added onto the air-core, to further reduce the propagation loss. The ITO relative permittivity is presented in Fig. S1-a, at different wavelengths, for bottom part of 250 C annealed ITO. We used a simplified homogenous property of ITO with the relative permittivity of and -0.0888 +0.584503i at 1550 nm, as well as 0.006033 +0.564856i at 1530 nm.

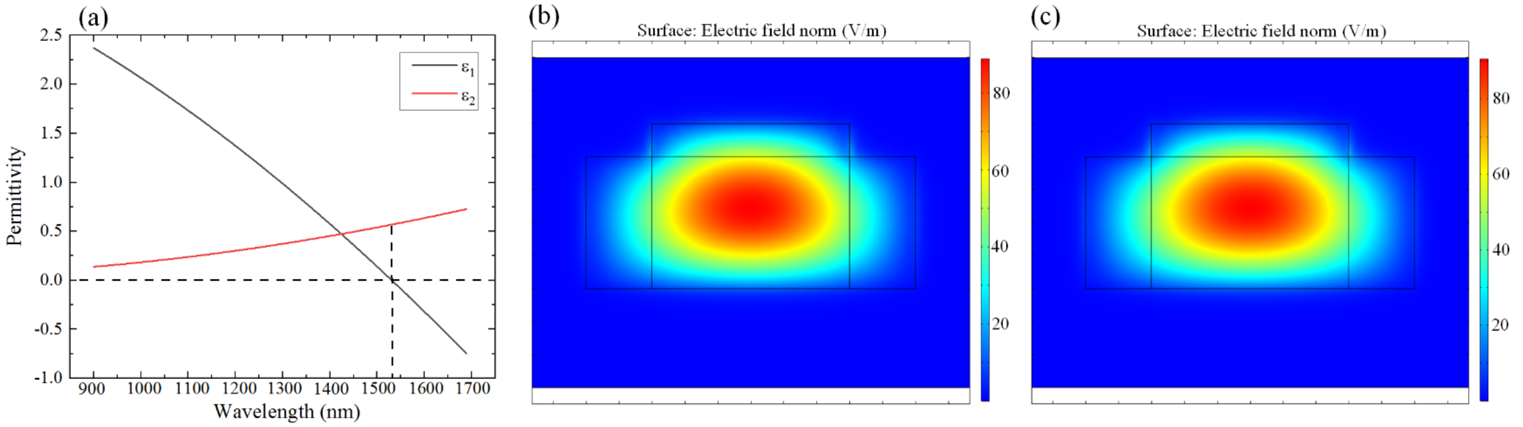

Supplementary Fig. S1. (a) Relative permittivity of ITO at different wavelengths. (b, c) Cross-sectional effective mode index found for air-core ENZ-cladding waveguides at 1550 nm and 1530 nm.

We used the FEM (COMSOL) to solve for effective mode index supported by the hollow-core structure at 1550 nm and 1530 nm. Figures S1-b and c represent the air-core waveguide TE mode at 1550 nm and 1530 nm, respectively. Effective indices are found to be 0.9859 - 0.0006979i, and 0.98614 - 0.00070736i, respectively.

The propagation loss can be calculated by the following equation:

$$L = 10\log_{10}(e^{-2*\frac{2\pi}{\lambda}*n_{eff}\cdot i\cdot Z}).$$

The propagation losses are 24.57 dB/mm and 24.91 dB/mm, respectively. This model provides an important insight towards the design of a practical ENZ waveguide.

S2. TEM Methods

TEM specimens were prepared by tripod polishing of film cross-sections followed by ion milling using a Gatan PIPS II ion polishing system. The specimens were characterized using a JEOL ARM200F TEM equipped with a windowless silicon-drift EDS detector and operated at 200 keV. Specimens were characterized using both conventional TEM imaging and STEM imaging combined with EDS mapping.

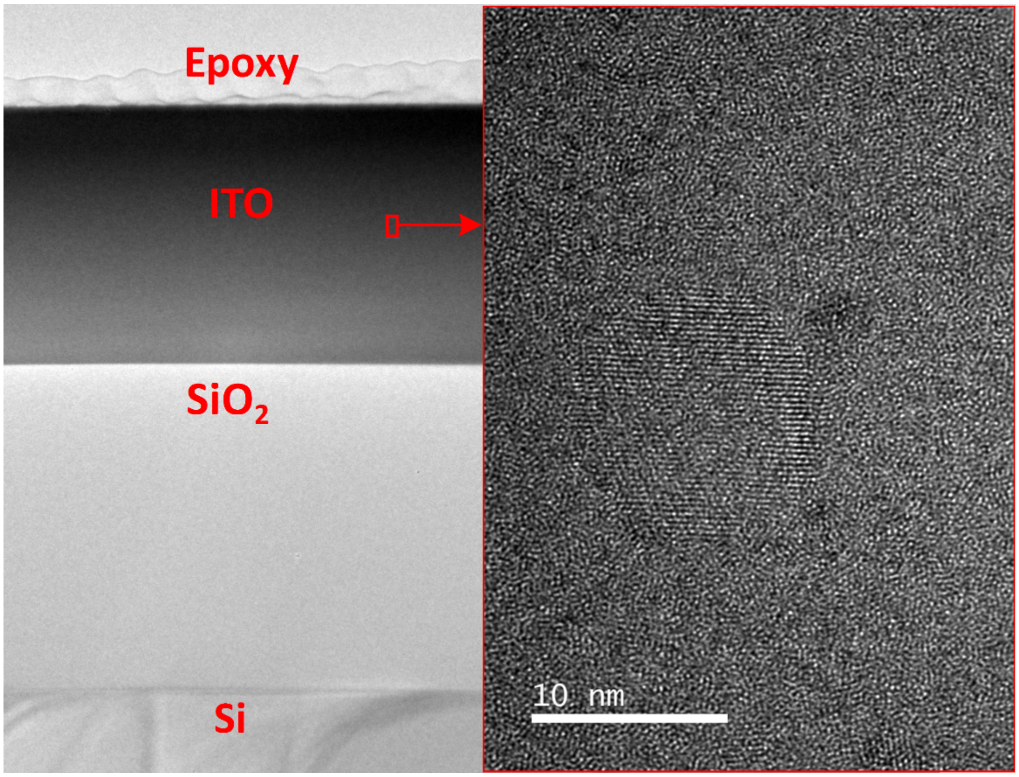

Supplementary Fig. S2. TEM images of non-annealed ITO metafilm.

Uniformly amorphous ITO has been observed experimentally for the non-annealed sample. A series of TEM images have been produced to verify on the non-annealed sample. Supplementary Fig. S2 is the low-magnification TEM image, and the inset is a high-resolution TEM image. Each image was recorded with e-beam of 200 keV, and it is immediately clear that the ITO film stack is uniformly amorphous and flat at both interfaces with SiO_2 and epoxy (details can be found in the Methods section). Rather, the bottom inset of Supplementary Fig. S2 closely presents the amorphous nature of ITO sample as-grown. As described in detail in Methods, a long process of ion milling was carried out to ensure the film transparency to TEM e-beam. Notice that the top inset micrograph of Supplementary Fig. S2 confirms the partial crystalline of the ITO film under high energy e-beam for an extended period of time (focusing on the local field for more than a few seconds), which also suggests the crystallization can be initiated the local irradiation of high energy beam. As shown in supplementary movie, in-situ electron-beam irradiation (EBI) has been applied onto ITO film without thermal annealing. Under 200 keV, ITO crystallization is observed along the direction of e-beam gun. Under high resolution TEM

image taken at real-time, the formation of ITO crystalline and its lattice distance is precisely-measured to be 1.013 nm. The real-time FFT has shown a complete crystallization process of ITO metafilm under e-beam irradiation.

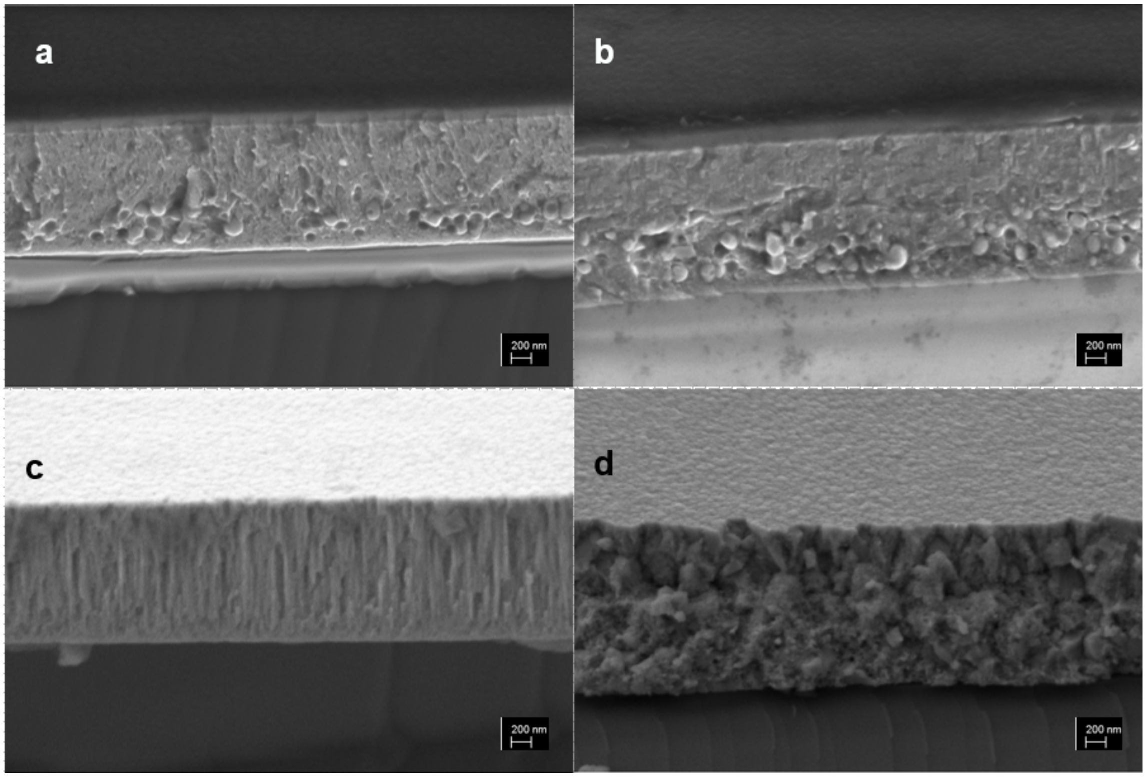

Supplementary Fig. S3. SEM images of ITO metafilm of (a) cap sample annealed 10 min at 300 C, (b) cap sample annealed 10 min at 375 C, (c) open surface sample annealed 10 min at 300 C, (d) open surface sample annealed 10 min at 375 C.

In addition to TEM cross-section scans, we have also acquired SEM cross-section scans of different ITO samples under different annealing conditions. Supplementary Fig. S3 shows a comparison of SiO_2 cap sample with open surface ITO sample. Both were investigated by the cross-section SEM to determine the polycrystalline along the growth direction. Starting at 275 C, we were able to observe the morphology of ITO polycrystalline change, as the bottom half starts to form bigger equaxed grains and top half starts to show columnar grains.

S3. Determining Thick Film Permittivity—Ellipsometer Modeling

The permittivity of the samples analyzed in this work was particularly challenging to estimate, because the samples consisted of multi-layer film stacks that included a thick layer of non-uniform ITO. Compounding on the difficulty imposed by the complexity of the samples, the permittivity of a film cannot be measured directly. Instead, we used a technique known as spectroscopic ellipsometry to measure the permittivity. In this technique, spectrally resolved polarization measurements of the light reflected from the sample under test are combined with advanced physical modeling to estimate the wavelength-dependent permittivity of multi-layer samples. We used a J. A. Woollam M-2000 spectroscopic ellipsometer to perform the ellipsometer measurements, and we used J. A. Woollam's associated modeling software, CompleteEASE, in order to fit a permittivity model to the measured reflection data. In this section, we provide further details regarding the modeling process used to obtain the permittivity spectra reported in the main text.

In general, a variety of physical mechanisms, such as band-gaps, color centers, and the Drude response of free-carriers result in resonances in the material's response to an incident electromagnetic field. The sum of these resonances yields the frequency-dependent permittivity. We used the CompleteEASE software to construct a physical model for the permittivity of the ITO layer according to the known resonances in the material's response to an incident electromagnetic field. The permittivity in ITO at wavelengths near 1550 nm is dominated by the Drude response of its free carriers. At shorter wavelengths, other resonances, such as the band-gap, are dominant; however, their contribution to the permittivity

at sufficiently long wavelengths can be modeled by the wavelength-constant parameter, ε_∞. As we discuss later, we chose to limit the wavelengths measured to the range between 900 and 1700 nm, such that it was sufficient to consider only the Drude model. Hence, the model for the frequency-dependent permittivity took the form

$$\varepsilon(E) = \varepsilon_\infty - \hbar^2/[\varepsilon_0\, \rho\, (\tau E^2 + i\hbar E)],$$

where E is the energy of the incident photons, $\hbar$ is the reduced Planck constant, ε_0 is the vacuum permittivity, ρ is the resistivity of the film, and τ is the scattering time of the free carriers. We note that the energy is related to the frequency by $E = \hbar\omega$, and the resistivity is related to the density of free carriers by $\rho = m^*/(Nq^2\tau)$, where m^* is the reduced mass of the free carriers, N is the free carrier density, and q is the elementary charge. So, the equation reported in the main text for the zero-crossing frequency of the real part of the permittivity can be derived from the above equation.

Aside from the physical model for the permittivity of the ITO layer, the CompleteEASE software took into account a variety of other parameters, including the thickness of the ITO layer, the thickness and permittivity of any other material layers of the sample, the sample's surface roughness, and several parameters to account for systematic uncertainties in the ellipsometer measurement. We physically measured the layer thicknesses and used known values for the permittivity of the silicon oxide layer in our sample. So, the free parameters in our model were the ITO layer resistivity, the ITO layer scattering time, and the high-frequency permittivity.

Our analysis of the TEM images indicated that the microstructure of the thick ITO films was not uniform; so, it was reasonable to hypothesize that the permittivity was not a constant in the growth direction. In particular, according to the physical picture outlined in the main text, we expected that the free carrier density and the scattering time varied because of a non-uniform density of oxygen vacancies and the non-uniform size of crystal grain boundaries, respectively. Indeed, we were unable to obtain a good fit to the measured data using models that treated the ITO as a uniform layer. Therefore, we used the graded layer feature of the CompleteEASE software to treat the 2μm ITO layer as a stack of 30 equally spaced slices, each of which had different values for the resistivity and scattering time. The values of these parameters in each slice was calculated according to a user-defined equation. In our case, we chose to parametrize the graded parameters with a two-segment linear model. This model corresponded to the microanalysis of the ITO films, which suggested that the material primarily consisted of two distinct regions, with the other regions being thin transition layers. Supplementary Fig. S4 shows an example of the graded resistivity as a function of the film depth. This data corresponds to the values used for the 350C-annealed sample. Notably, the two-segment linear model allows for different slopes of grading to be used in the two distinct regions. For the 350C-annealed sample, we found that the resistivity was nearly constant in the bottom half of the ITO layer, whereas it showed a significantly steeper slope in the top half of the ITO layer.

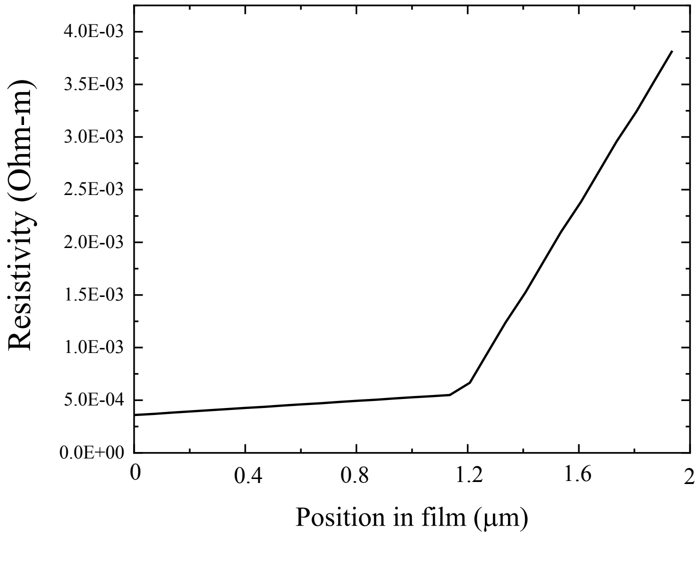

Supplementary Fig. S4. Resistivity of the ITO film stack, from bottom to top.

We determined the quality of each fit based on the mean-squared error (MSE) reported by the CompleteEASE software. MSE is useful for determining the quality of fit, since reducing the MSE indicates a better fit. All of the fits reported in the main text had MSE below 100. However, we note that the MSE is not a good metric to use for absolute

comparisons between different models. For example, we initially considered an expanded range of wavelengths, 370 nm – 1700 nm. We used Tauc-Lorentz and Gaussian oscillators to model the bandgap of the ITO film. However, we found that the MSE was dominated by the quality of fit at shorter wavelengths, and consequently, the fit in the lower wavelength region was not very good. Consequently, we decided to restrict the wavelengths considered to the range 900 nm – 1700 nm, so that we could better fit the data at the wavelength range of interest.

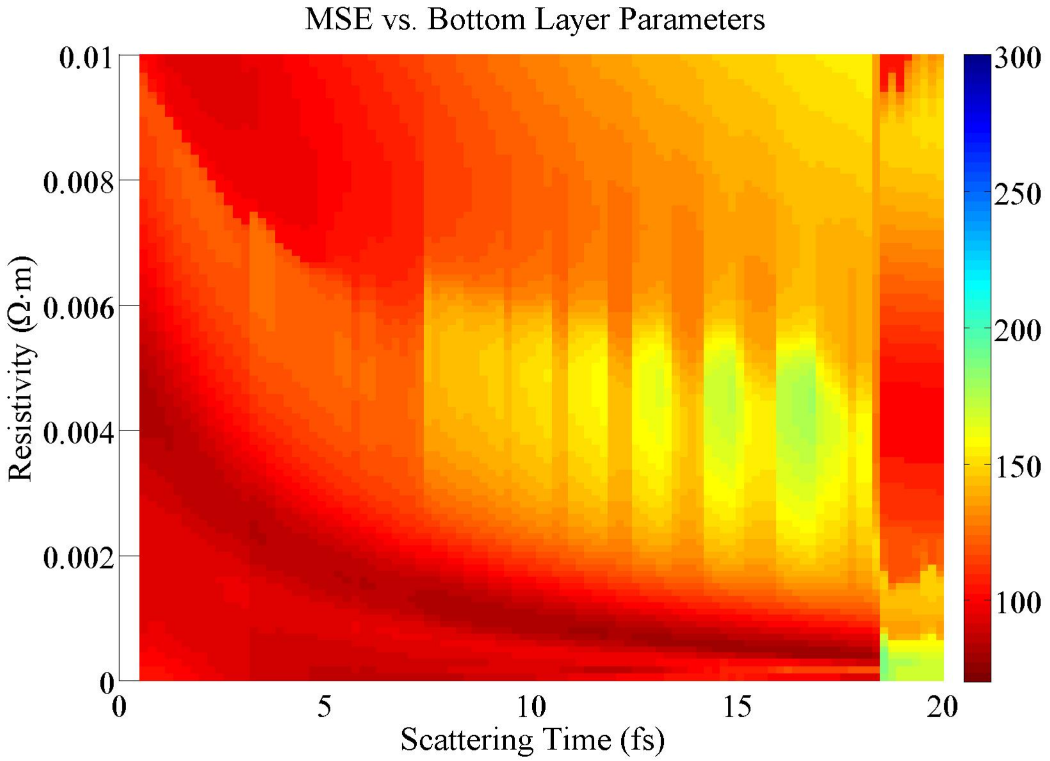

Supplementary Fig. S5. Drude model analysis: finding the optimum fitting by varying resistivity and scattering time.

Supplementary Fig. S5 shows the relationship between the resistivity of the ITO film and the scattering time under various fitting model. When scanning the only two parameters from Drude model (resistivity and scattering time), we are able to have a better understanding of the ellipsometry model of the ITO bottom layer. The mean standard error (MSE) represents how reasonable the fitting is by adopting different fitting parameters. Supplementary Fig. S5 is showing a clear trend that the resistivity parameter is dominating the Drude model, as we can only get a reasonable fitting within resistivity range under 10^{-3} Ω·m, while scattering time can vary from 12 to 18 fs.

[S1] M. H. Javani and M. I. Stockman, “Real and Imaginary Properties of Epsilon Near Zero Materials,” Phys. Rev. Lett., vol. 117, 107404, 2016.
